# Between the school and the screen: how digital informal learning shapes Chinese novice physical education teachers' identity

**DOI:** 10.3389/fpsyg.2026.1767468

**Published:** 2026-04-16

**Authors:** Qingyuan Zhou, Jiajun Jiang, Zhihua Yin, Yaowu Liu

**Affiliations:** College of Physical Education and Health, East China Normal University, Shanghai, China

**Keywords:** Chinese novice physical education teachers, digital informal learning, field and habitus theory, identity construction, reflective thematic analysis, social constructivist theory

## Abstract

**Objectives:**

While digital informal learning has gained increasing attention in teacher development research, limited studies have examined its role in the identity construction of Chinese novice physical education teachers. This study explored how digital informal learning informs identity negotiation processes within the intersecting contexts of “school” and “screen,” providing new empirical and theoretical insights into early professional development in the digital age.

**Methods:**

This qualitative study employed individual semi-structured interviews and focus groups with 25 novice physical education teachers in China, supplemented by teacher reflection journals, social media posts, and digital interaction records. The data were analyzed using reflexive thematic analysis.

**Results:**

Three interconnected themes were identified: (1) digital informal learning as an orientation space amid role ambiguity, (2) experimenting with teaching practices through digital informal learning, and (3) articulating professional identity through digital community participation.

**Conclusion:**

Findings indicate that digital informal learning enables novice physical education teachers to interpret practice, develop pedagogical judgment, and situate themselves within broader professional communities, through which professional identity is negotiated across school and digital contexts, while also generating tensions as these contexts operate according to different practical and evaluative logics.

## 1 Introduction

In the digital era, digital platforms such as social media (Carpenter et al., 2025), short video services (Beck and Riddle, 2024), podcasts, and online communities (King and Watt, 2024; Zhong and Craig, 2020) have increasingly emerged as essential venues through which physical education teachers articulate their perspectives, engage in peer observation, and construct professional identities. Against this backdrop, digital informal learning, which refers to spontaneous and practice-oriented activities undertaken by physical education teachers within digital media contexts beyond formal institutional structures (Shin and Park, 2025), provides novel avenues for professional identity development and formation. In contrast to formal training—which prioritizes standardized knowledge and technical conventions (Donitsa-Schmidt and Zuzovsky, 2018)—digital informal learning capitalizes on immediacy, adaptability, and interactivity (Le et al., 2021), thereby offering physical education teachers a distinctive route toward professional recognition and identity formation. This phenomenon indicates that the construction of professional identity among physical education teachers is no longer confined exclusively to the formal institutional setting of the “school,” but is also experiencing a gradual transformation within the digitally mediated realm symbolized by the “screen.”

As the professional learning of physical education teachers progressively extends from traditional school settings to digital environments, the influence of digital informal learning on novice physical education teachers warrants particular scholarly attention. In comparison with experienced teachers, who have established relatively stable teaching expertise and professional judgment, novice teachers in the early stages of their careers tend to rely more heavily on digital informal learning as a primary reference for classroom management, emotional regulation, and professional expression when confronted with the complexity and uncertainty of teaching situations (Westerlund and Eliasson, 2022). Through sustained observation, communication, and interaction on digital platforms, novice physical education teachers not only acquire instructional strategies and practical experience (Zhong and Craig, 2020), but also progressively develop an understanding of “what it means to be a physical education teacher,” thereby shaping the construction of their professional identity. Therefore, examining the identity construction of novice physical education teachers from the perspective of digital informal learning contributes to revealing how digital spaces intersect with school contexts to co-construct the process of early professional development. This approach also demonstrates strong group representativeness, practical relevance, and theoretical significance.

In recent years, scholarly research on “digital informal learning” and “teacher identity construction” has expanded rapidly; however, notable limitations remain with respect to disciplinary coverage, career stages, and research orientation. First, with regard to disciplinary coverage, existing studies have primarily concentrated on fields such as English (Liu and Wang, 2024) and STEM (Liu et al., 2024), whereas physical education teachers have received comparatively limited scholarly attention. Second, in relation to career stages, prior research has largely focused on experienced teachers (Brooks and McMullen, 2020) or pre-service teachers (Hyndman and Harvey, 2020), while comparatively little attention has been paid to novice physical education teachers whose professional identities remain in formative and adaptive phases. Third, with respect to research orientation, theoretical scholarship has highlighted the generative nature of physical education teacher identity, arguing that such identity is constructed through continuous negotiation and reflective practice embedded within specific sociocultural contexts (Anwar et al., 2025; Shen et al., 2022). In this sense, when teachers face situations such as instructional dilemmas, occupational pressures, and role expectations, their adjustments to their own professional positioning and understanding of the teacher role constitute an important manifestation of the construction of professional identity (Beijaard et al., 2004; Hanna et al., 2019). At the practical level, empirical studies have increasingly examined how physical education teachers participate in self-directed professional learning via digital spaces, including social media, online communities, and various digital platforms (Ko et al., 2023; McNamara et al., 2022). At the outcome level, existing research has explored the potentially supportive, challenging, and uncertain effects of digital informal learning on the professional development of physical education teachers (Alcolea Lozano and Camacho-Miñano, 2024; Li and Casey, 2026). However, from a process-oriented perspective, existing studies have not yet provided sufficient analysis of the specific mechanisms through which digital informal learning operates in the identity construction of novice physical education teachers, particularly in terms of its interaction with school-based work contexts and its joint influence on teachers' role perceptions and professional identity development (Hyndman and Harvey, 2020).

Therefore, against the backdrop of digital informal learning increasingly constituting an integral component of teachers' professional practice, and in light of existing research gaps concerning the context of physical education, the career development stages of novice teachers, and the process of identity construction, this study focused on novice physical education teachers in China, defined as those with less than 2 years of teaching experience, and examined how they perceive and engage in digital informal learning. Grounded in a social constructivist perspective and informed by Bourdieu's theoretical framework of “field” and “habitus,” this paper further investigates how digital informal learning, as a practical resource embedded in teachers' everyday professional practices, shapes the identity negotiation processes of novice physical education teachers within the intersecting fields of “school” and “screen.” Consequently, this study provides new empirical evidence and theoretical implications for understanding the early professional development of physical education teachers in the digital age.

## 2 Theoretical framework

Social constructivist theory posits that identity is not an inherent attribute of the individual, but is instead gradually constructed through interaction, participation in social practices, and reflection on experiences within specific social contexts (Berger and Luckmann, 1966; Vygotsky, 1978). In other words, the professional identity of novice physical education teachers is generally conceptualized as an ongoing developmental process, in which they continuously interpret and adjust their teaching roles through interactions with students, colleagues, and the broader professional community. Therefore, in this study, social constructivist theory provides an analytical lens for understanding how novice physical education teachers, based on their practical experiences and interactive processes, gradually construct and develop their professional identities through communication and observation in both school settings and digital platforms.

Bourdieu (1977, 1990) theory of fields and habitus posits that society comprises multiple fields, each characterized by relatively stable rules, resources, and forms of capital, and that the habitus developed by individuals within these fields shapes their cognition and behavior (Bourdieu, 1993, 1996). From this perspective, the professional identity of novice physical education teachers is not only gradually constructed through interaction, but also shaped within specific institutional contexts and professional norms. For novice physical education teachers, the transition from teacher training institutions to K−12 schools, while simultaneously engaging in informal learning on digital platforms, entails movement across different educational fields. The distinct practical logics and professional expectations inherent in these fields exert a significant influence on novice teachers' professional understanding and identity development.

Taken together, social constructivist theory provides a lens for understanding how novice physical education teachers construct their professional identities through specific interactions and practices, while Bourdieu's theories of field and habitus illuminate the educational fields in which this identity construction occurs and their underlying institutional logics. By integrating these two approaches, this study adopts an analytical perspective that bridges micro- and macro-levels as well as internal and external dimensions. This enables a more comprehensive understanding of the relationships between individual dilemmas and social fields, as well as between internal experiences and institutional contexts, thereby elucidating the processes through which novice physical education teachers in China negotiate and develop their professional identities across school settings and digital informal learning environments.

## 3 Context

In the Chinese educational context, novice physical education teachers frequently encounter multifaceted professional challenges. First, educational resources have historically been concentrated on core subjects such as Chinese, mathematics, and English—central components of the high school and university entrance examinations—whereas physical education, which is excluded from these high-stakes assessments (Ma, 2013), continues to be marginalized in terms of curriculum emphasis, instructional support, and professional recognition (Yang et al., 2025). Second, pre-service training programs for physical education teachers remain predominantly theory-oriented, with the majority of students completing only a single semester of practicum experience. This limited exposure to practical teaching renders them insufficiently prepared to meet the multifaceted demands of authentic classroom environments (Han, 2023). Post-employment support mechanisms for novice physical education teachers remain insufficient, resulting in professional adaptation processes that rely heavily on personal initiative and experiential accumulation (Yan et al., 2025). Furthermore, disparities in regional development contribute to pronounced differences across schools in terms of educational infrastructure, resource distribution, and students' physical fitness baselines (Wang et al., 2025), thereby imposing elevated demands on novice teachers' adaptability and pedagogical flexibility.

Against this backdrop, digital informal learning has increasingly emerged as a critical avenue through which novice physical education teachers in China access pedagogical resources, acquire practical experience, articulate professional viewpoints, and seek emotional affiliation. Digital platforms including WeChat Official Accounts, Douyin, Xiaohongshu, Bilibili, and Zhihu have aggregated extensive physical education–related instructional content, such as teaching videos, curricular case analyses, and professional discourse (Li and Casey, 2026), thereby constructing a learning environment that transcends geographic constraints, institutional boundaries, and disciplinary identities.

## 4 Methodology

This study employed a qualitative research design, informed by interpretive description, to examine how digital informal learning shapes the professional identity construction of novice physical education teachers in China. This design was used because the study aimed to explore how participants make sense of their engagement in digital informal learning and how these experiences contribute to the development of their professional identities. Consistent with this orientation, the study focused on capturing participants' subjective understandings and the processes through which identity is negotiated across school and digital contexts. This approach is well-suited to research on teacher identity, which emphasizes the dynamic, context-dependent, and meaning-oriented nature of professional development.

### 4.1 Participants

This study recruited 25 novice physical education teachers from mainland China, aged 22–26 years (*M* = 23.92, SD = 1.00), all possessing 1–2 years of teaching experience. The sample included 15 male and 10 female participants, all of whom were employed in public schools at the time of the study. Their teaching assignments spanned elementary (Grades 1–6, *n* = 6), junior secondary (Grades 7–9, *n* = 7), and senior secondary levels (Grades 10–12, *n* = 12). Geographically, the participants' schools were distributed across East, Central, South, Southwest, Northeast, and North China. The sample comprised 21 participants from urban schools and four from suburban institutions. Participants' weekly teaching loads ranged from 8 to 22 physical education classes (*M* = 14.48, SD = 4.10), reflecting a phase of heavy teaching responsibilities early in their careers while still accumulating professional experience (Table 1 for details).

**Table 1:** Basic information of the interviewee.

ID	Gender	Age	Grade taught	Teaching year(s)	School type	Teaching location	Weekly teaching hours
T01	Female	24	Grades 1–6	1	Suburban Public	East China	15
T02	Male	24	Grades 7–9	1	Urban Public	Southwest	15
T03	Female	24	Grades 10–12	1	Urban Public	East China	12
T04	Male	23	Grades 10–12	1	Urban Public	Northeast	8
T05	Male	23	Grades 7–9	1	Urban Public	East China	21
T06	Male	22	Grades 10–12	1	Urban Public	Central China	10
T07	Female	23	Grades 1–6	1	Urban Public	East China	18
T08	Female	24	Grades 1–6	1	Urban Public	Southwest	16
T09	Female	25	Grades 7–9	1	Urban Public	North China	12
T10	Male	24	Grades 7–9	1	Urban Public	Central China	9
T11	Male	23	Grades 10–12	1	Urban Public	Central China	10
T12	Female	22	Grades 1–6	1	Suburban Public	Central China	12
T13	Male	23	Grades 7–9	1	Urban Public	North China	16
T14	Female	24	Grades 10–12	1	Urban Public	East China	20
T15	Female	25	Grades 1–6	2	Urban Public	East China	17
T16	Male	26	Grades 1–6	2	Urban Public	Southwest	22
T17	Male	24	Grades 10–12	2	Urban Public	East China	12
T18	Female	23	Grades 10–12	2	Urban Public	East China	21
T19	Male	25	Grades 7–9	2	Suburban Public	East China	16
T20	Female	25	Grades 10–12	2	Urban Public	South China	10
T21	Male	25	Grades 10–12	2	Urban Public	East China	20
T22	Male	24	Grades 10–12	2	Urban Public	East China	12
T23	Male	24	Grades 7–9	2	Urban Public	East China	12
T24	Male	24	Grades 10–12	2	Suburban Public	East China	12
T25	Male	25	Grades 10–12	2	Urban Public	Southwest	14

Sample recruitment employed a strategy that combined purposive sampling and snowball sampling. Purposive sampling was employed to identify novice physical education teachers who met the study's criteria, thereby ensuring that participants reflected the career development stage and learning contexts of interest to the research. Participants were required to meet the following conditions: (1) be in the early stages of their careers, with 1–2 years of teaching experience; (2) have completed at least one full teaching cycle and possess relevant teaching experience; and (3) have encountered or utilized digital platforms for informal learning during their daily teaching or professional development. The initial sample was drawn from a network of early-career teachers established by the research team between 2023 and 2024 through teacher training programs and student teaching supervision. Subsequently, the sample scope was expanded through school recommendations and teacher referrals in order to enhance the diversity of sample sources. The sample size was determined in accordance with the principle of theoretical saturation in qualitative research (Rahimi and Khatooni, 2024). By the 22nd participant, the research team observed that the core themes had stabilized, with no new concepts or interpretive dimensions emerging. To further validate the robustness of this saturation, the research team conducted interviews with an additional three teachers, resulting in a final sample of 25 participants.

With regard to research ethics, all participants were informed—via email or face-to-face meetings prior to the interviews—of the study's objectives, data usage protocols, and anonymization procedures, and subsequently signed written informed consent forms. Participants retained the right to withdraw from the study at any point throughout the research process. Personal and institutional identifiers were anonymized during data processing and presentation to protect participants' privacy and ensure their freedom of expression.

### 4.2 Data collection

The data for this study were primarily collected through interviews over a 3-month period (May–July 2025). The interviews were conducted in two formats: individual semi-structured interviews and focus groups. The decision to employ both formats was based on their complementary strengths in data generation: individual semi-structured interviews facilitated a deeper understanding of novice physical education teachers' personal experiences, cognitive shifts, and perceptions of identity during their early career engagement in digital informal learning, whereas focus groups promoted comparative analysis and collective reflection through peer interaction, thereby enabling perspectives that are difficult to articulate in one-on-one interviews to emerge during group discussions.

To progressively deepen the understanding of the research topic, the interviews were conducted in three phases. The first phase aimed to gather preliminary descriptions of novice physical education teachers' experiences with digital informal learning, including 10 semi-structured interviews and two focus groups comprising six participants each. The second phase was conducted following a preliminary analysis and reflection on the data from the first phase, focusing on further exploration of key experiences and recurring issues identified across the interviews. In this phase, the 22 teachers who had previously participated were reorganized into 12 individual semi-structured interviews and two focus groups comprising five participants each. The third phase involved recruiting three additional novice physical education teachers for individual semi-structured interviews to further enrich the dataset, building on the findings from the first two phases. Overall, each participant took part in one to two interviews, with each session lasting approximately 30–60 min. The interviews focused on novice physical education teachers' specific experiences with digital informal learning during the early stages of their careers, the associated impacts, and their understandings of professional identity. The specific interview topics are presented in Table 2. The format of the interviews was flexibly determined based on participants' schedules and geographic locations, with face-to-face interviews prioritized whenever feasible. When in-person interviews were not feasible, online interviews were conducted via the Tencent Meeting platform. During the interviews, the research team sought to foster an open and egalitarian communicative atmosphere to encourage participants to express themselves fully and engage in meaningful interaction. Concurrently, the researchers engaged in ongoing reflexivity throughout the interviews to critically assess their influence on the questioning strategies and the nature of the interactions. All interviews were audio-recorded following the acquisition of written informed consent and were transcribed verbatim by the research team within 48 h to ensure data integrity and contextual accuracy.

**Table 2:** Interview content.

No.	Interview topics
(1)	Emotional experiences, role expectations, and practical dilemmas faced by novice physical education teachers during their initial employment period
(2)	Motivations and opportunities that lead novice physical education teachers to begin engaging with digital informal learning
(3)	Strategies for selecting, applying, and adapting online resources as novice physical education teachers gradually accumulate experience
(4)	Changes and impacts perceived by novice physical education teachers through their continued engagement in digital informal learning
(5)	Novice physical education teachers' understandings and reflections on changes in professional identity as they navigate between “school” and “screen”

To further enrich the data sources and enhance the depth of the research interpretation, this study also collected supplementary materials, including teaching reflection journals, social media posts, and digital interaction records from participants' first 1–2 years of employment, all of which were voluntarily provided. In total, 43 reflection journals from 15 participants documented their teaching experiences and emotional responses, while 37 social media posts from 13 participants captured their public expressions of teaching practices in digital contexts. In addition, 51 digital interaction records from 17 participants—including browsing histories, saved content, and online exchanges—provided insight into their engagement with digital informal learning. All materials were collected within the same time frame as the interviews and were integrated into the coding process alongside interview transcripts. Rather than being treated as independent datasets, these materials served to triangulate self-reported accounts and to provide contextual and behavioral evidence, thereby strengthening the depth and credibility of the analysis.

### 4.3 Data analysis

Data analysis was conducted using reflexive thematic analysis, following the six-phase procedure proposed by Braun and Clarke (2006, 2021). This approach conceptualizes themes as interpretive outcomes generated through the researcher's active engagement with the data. NVivo 14.0 (QSR International Pty Ltd, Doncaster, Victoria, Australia) was employed for data management and for tracking coding processes to ensure analytical rigor, transparency, and traceability. Throughout each phase of analysis, researchers maintained reflective notes, critically examining their interpretive positions, underlying assumptions, and analytical trajectories. This reflexive approach ensured that theme generation remained grounded in iterative dialogue with the data and sustained critical engagement.

The analytical procedure consisted of the following six phases: (1) familiarization with the data: all interview recordings were transcribed verbatim and read repeatedly to facilitate immersion in the data. Simultaneously, supplementary materials were systematically organized. During this phase, the researcher identified key expressions, emotional cues, and recurring narrative patterns by drawing on prior understanding of novice physical education teachers' work contexts and digital platform environments. An initial holistic understanding of the dataset was developed through the documentation of intuitive impressions and analytical memos. (2) Initial coding: open coding was conducted based on participants' direct statements, focusing on emotional responses, learning practices, and role perceptions. This phase emphasized the researcher's interpretive engagement, acknowledging that the same data segment could be interpreted differently across contexts. Through iterative returns to the data and the comparison of multiple interpretive possibilities, the analytical focus gradually crystallized. (3) Theme development: codes sharing similar meanings or discursive functions were clustered to form preliminary themes. Through continuous review of the raw data and reflexive consideration of the researcher's interpretive stance, emerging patterns of identity construction among novice physical education teachers within digital informal learning contexts were distilled. (4) Reviewing and refining themes: preliminary themes were systematically examined in terms of their internal coherence and explanatory capacity. Themes with ambiguous meanings, overlapping interpretations, or limited relevance to the research questions were refined through processes of merging, splitting, or renaming to enhance explanatory validity. (5) Defining the thematic structure: following multiple rounds of revision, the logical relationships among themes were organized and integrated, resulting in three core themes with progressive interconnections. These themes both represent novice physical education teachers' digital informal learning practices and elucidate how their professional identities gradually emerged and were reconfigured through phases of confusion, experimentation, and positioning. (6) Final analysis and reporting: representative and analytically rich data excerpts were integrated into the analytical narrative, accompanied by interpretive discussion grounded in relevant theoretical perspectives.

### 4.4 Trustworthiness

Within the framework of reflective thematic analysis, the credibility of the research is grounded in the transparency and methodological rigor of the analytical process, as evidenced by ongoing reflexivity, iterative theme refinement, and sustained engagement with the data by the research team (Strunel and Cordoba, 2025). Specifically, the first and second authors (ZQY and JJJ) engaged in multiple rounds of collaborative discussions on preliminary themes, with a focus on delineating thematic boundaries and internal coherence to ensure that the constructed themes captured the complexity and polysemy inherent in the data. Preliminary findings were subsequently reviewed by the third author (YZH) to provide external feedback and validation. The feedback contributed to the expansion of interpretive pathways and further deepened the team's understanding of the dataset. Building upon this foundation, the research team continuously revisited the original dataset and accompanying analytical memos throughout the process of initial coding and theme development. This reflexive process was intended to identify potential interpretive biases and, where necessary, revise theme structures and analytical content to ensure coherence and depth in interpretation.

## 5 Results

**Figure 1:**
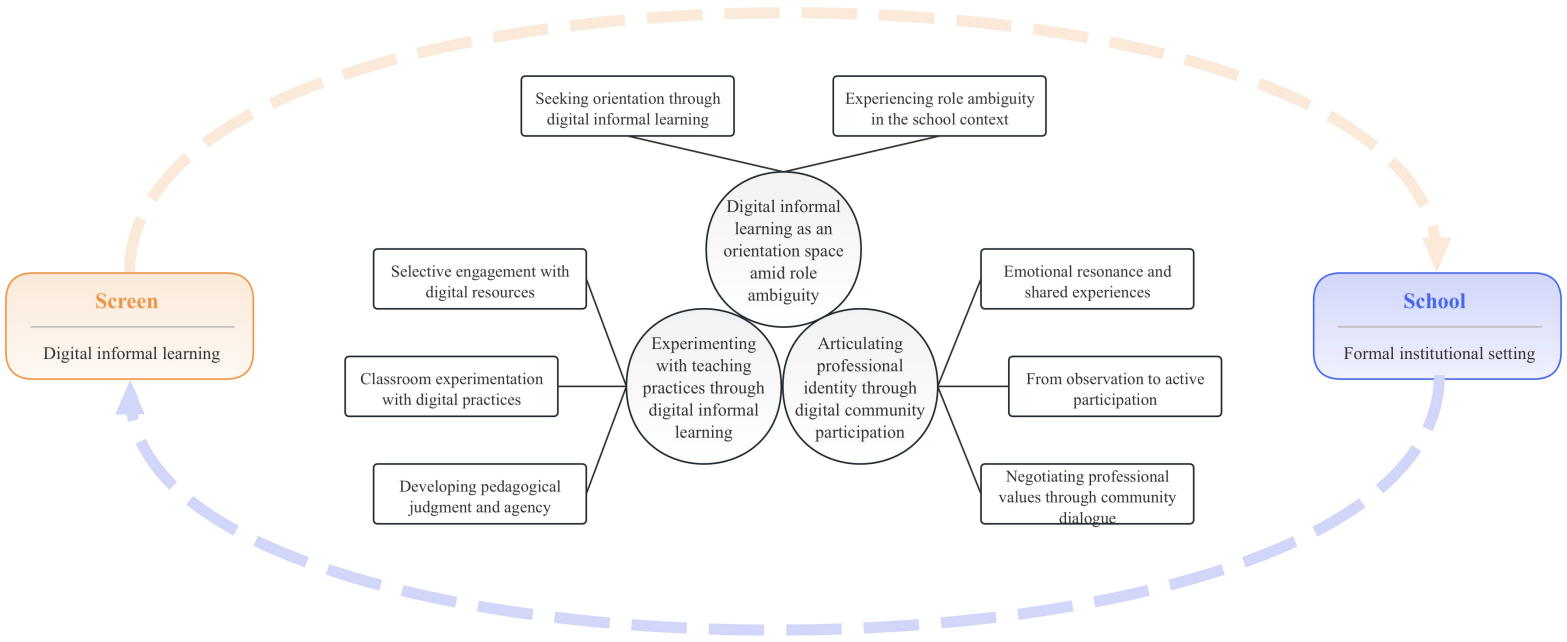
Final themes and sub-themes.

Based on interview transcripts, teacher reflection journals, social media posts, and interaction records derived from digital learning engagements, the study identified three core themes (see Figure 1).

### 5.1 Theme I: digital informal learning as an orientation space amid role ambiguity

Across the dataset, novice physical education teachers frequently described their early teaching experiences using expressions such as “confused,” “at a loss,” and “figuring things out on my own.” These recurring descriptions suggest that entering the profession did not immediately provide a clear understanding of how to enact the role of a physical education teacher in everyday school practice. Instead, participants often experienced a period of role ambiguity during which their professional identity remained unsettled. In this context, digital informal learning gradually emerged as an orientation space through which teachers attempted to interpret their early professional experiences.

#### 5.1.1 Experiencing role ambiguity in the school context

Within the school setting, formal induction arrangements such as mentorship programs and initial training were intended to support novice teachers' transition into professional practice. However, participants' accounts suggest that these mechanisms often provided limited guidance for addressing the practical and situational challenges of physical education teaching.

*For example, one teacher reflected on the limited feedback received through the school's mentorship system:*

“*During my first two months on the job, our school's ‘mentorship program' mainly involved observing a few classes before I began teaching independently. There was no feedback provided after my lessons. (T03, individual semi-structured interview)”*

*Similarly, another participant described the difficulty of translating formal training into everyday classroom practice:*

“*Although I learned certain content during the training in my first three months, applying it effectively in classroom practice remained difficult. (T17, individual semi-structured interview)”*

Beyond institutional arrangements, the inherently dynamic nature of physical education lessons further intensified teachers' uncertainty. Compared with classroom-based subjects, physical education teaching is strongly shaped by contextual factors such as playground space, weather conditions, equipment availability, and student dynamics.

*As one teacher noted in a reflection journal:*

“*The playground is often shared with other classes during lessons, which makes it impossible to implement the originally planned teaching activities—the available space is simply too limited. (T08, reflection journals)”*

Under such conditions, novice teachers often found it difficult to evaluate whether their teaching strategies were appropriate or effective. As a result, the early stage of professional practice was frequently characterized by uncertainty regarding both classroom decision-making and professional expectations.

#### 5.1.2 Seeking orientation through digital informal learning

Faced with these uncertainties in the school context, many novice teachers began turning to digital platforms as an additional point of reference. Rather than functioning as a formal training system, digital informal learning provided a flexible and accessible space where teachers could observe how other practitioners described and addressed similar teaching situations. Through browsing teaching videos, lesson demonstrations, and experience-sharing posts, teachers attempted to interpret their own classroom experiences by comparing them with those of others.

*Several participants described using online resources in this way:*

“*When I encounter problems in class, I sometimes search online to see how other physical education teachers handle similar situations. (T06, digital interaction records)”*

“*Sometimes I watch teaching videos online to see how other teachers organize their lessons, because I want to know whether my approach is reasonable. (T11, digital interaction records)”*

Through exposure to these online discussions and resources, teachers gradually recognized that many of the difficulties they encountered were widely shared among early career teachers. In this sense, digital informal learning did not primarily function as a space for developing new teaching strategies. Instead, it served as an orientation space through which novice teachers could interpret their early professional experiences. By situating their classroom challenges within a broader community of practitioners, participants began to make sense of their roles between the expectations of the school environment and the experiences shared within digital spaces.

### 5.2 Theme II: experimenting with teaching practices through digital informal learning

As novice physical education teachers became more familiar with digital platforms and accumulated classroom experience, their engagement with digital informal learning gradually evolved. While digital platforms initially served as spaces for observing examples and gathering teaching ideas, teachers increasingly began to experiment with instructional practices by adapting and refining digital resources in response to their own classroom contexts. In this process, digital informal learning shifted from being primarily a source of inspiration to becoming a space that supported ongoing pedagogical experimentation.

#### 5.2.1 Selective engagement with digital resources

During their initial engagement with digital platforms, novice physical education teachers often relied on online materials—such as lesson demonstrations, instructional videos, and experience-sharing posts—to gain teaching ideas. However, as teachers accumulated more classroom experience, many began to recognize that examples encountered online could not always be directly transferred into their own teaching environments. Differences in grade levels, student characteristics, and school conditions often required teachers to engage with digital resources more selectively.

*Participants described how their platform use gradually became more targeted and purposeful:*

“*Strategies for managing students of different age groups vary. Since I teach middle school students, I specifically search for ‘How to address poor discipline in middle school physical education classes.' (T05, digital interaction records)”*

“*When searching for resources on platforms like Douyin and Xiaohongshu, I will select content that genuinely aligns with our school's specific circumstances, rather than simply copying teaching materials directly from these platforms. (T07, reflection journals)”*

These accounts suggest that teachers increasingly treated platform content as reference material rather than ready-made solutions. Instead of focusing on whether a lesson looked appealing or widely shared online, they began to pay closer attention to whether the material aligned with their own students, classroom dynamics, and instructional goals.

#### 5.2.2 Classroom experimentation with digital practices

As teachers became more familiar with their classroom contexts, they began to test and adjust digital teaching ideas through repeated classroom implementation. Rather than following lesson plans found online step by step, participants described modifying activities, adjusting difficulty levels, and refining instructional strategies in response to students' reactions.

*Several teachers reflected on this process of adapting digital teaching ideas:*

“*I think this activity is well designed, but the difficulty level may not be entirely appropriate for my students. Later, I used tools such as ChatGPT or DeepSeek to help me adjust the activity steps. (T15, focus group)”*

“*At first, I followed the lesson plans I found online. However, as I taught the same lesson repeatedly, I began adjusting it in response to students' immediate reactions. Gradually, I found that it worked better than before. (T10, focus group)”*

These experiences illustrate how digital informal learning became intertwined with teachers' ongoing cycles of classroom experimentation. Instead of adopting online practices directly, teachers tested, revised, and adapted these ideas through repeated teaching attempts. At the same time, teachers sometimes found that teaching ideas which appeared promising online did not produce the expected results in their own classrooms:

“*Some instructional videos look quite appealing, but when I actually try them in class, the results are not as effective as expected, and students do not respond as positively as I imagined. (T02, individual semi-structured interview)”*

Such mismatches between digital resources and classroom realities often prompted further adjustment and reflection, reinforcing the experimental nature of teachers' engagement with digital materials.

#### 5.2.3 Developing pedagogical judgment and agency

Through repeated cycles of selection, experimentation, and revision, novice teachers gradually developed stronger pedagogical judgment in their engagement with digital resources. Rather than relying uncritically on platform-generated content, they increasingly evaluated, combined, and redesigned materials in ways that suited their own instructional contexts. In this process, digital platforms ceased to function merely as repositories of ready-made teaching solutions. Instead, they became supportive tools through which teachers compared different instructional approaches, integrated ideas from multiple sources, and refined their own teaching strategies.

*For example, one teacher shared a social media post reflecting on the preparation of her first demonstration lesson:*

“*Yay! Finally finished my first onboarding session. I scoured tons of resources online, watched countless high-quality lesson videos, and incorporated insights from other teachers along with my own reflections. Thankfully, it turned out pretty well in the end! (T20, social media post)”*

*The materials accompanying this post—including screenshots of instructional videos from different platforms and handwritten lesson notes—illustrated how teachers actively synthesized resources from multiple sources while developing their own teaching plans*.

These practices suggest that digital informal learning increasingly supported the development of pedagogical agency among novice teachers. Through ongoing experimentation and reflection, teachers began to rely less on direct imitation and more on their own professional judgment when deciding how digital resources could be adapted to support their instructional goals.

### 5.3 Theme III: articulating professional identity through digital community participation

As novice physical education teachers became more familiar with digital platforms and accumulated classroom experience, their engagement with digital informal learning gradually evolved. While digital platforms initially served as spaces for observing examples and gathering teaching ideas, teachers increasingly began to experiment with instructional practices by adapting and refining digital resources in response to their own classroom contexts. In this process, digital informal learning shifted from being primarily a source of inspiration to becoming a space that supported ongoing pedagogical experimentation.

#### 5.3.1 Emotional resonance and shared experiences

During the early stages of their teaching careers, many novice teachers described experiencing strong emotional resonance when encountering posts or videos shared by other teachers online. These experiences often mirrored their own classroom challenges, providing a sense of emotional comfort and reducing feelings of isolation.

*Participants recalled how encountering such content helped them realize that the difficulties they faced were not unique to them:*

“*When I first started working, I came across a video posted by another teacher describing students lining up in disarray and misbehaving in class—exactly what I was experiencing. After watching it, I almost cried with relief. (T18, focus group)”*

Similarly, one participant described repeatedly encountering social media posts shared by teachers who had entered the profession in the same year. These posts frequently documented everyday experiences such as consecutive classes, intensive lesson preparation, and working late to prepare for the next day's teaching.

*Reflecting on these posts, the participant explained:*

“*I truly felt their struggle. We were all going through the same thing at that time. (T04, social media post)”*

Through such encounters, teachers began to reinterpret their individual teaching struggles as part of a broader shared experience among early-career teachers. Digital platforms thus provided a space where novice teachers could collectively make sense of the challenges associated with entering the profession.

However, this process of comparison could also generate subtle pressure. As teachers continued to observe the progress of their peers online, some began to evaluate their own teaching in relation to others' perceived development:

“*At first, I thought I was performing very poorly. However, after observing others, I realized that everyone was learning on the job. But after seeing so much over time, I sometimes wonder—everyone else seems to be gradually finding their footing. Should I not be able to do the same? (T11, individual semi-structured interview)”*

#### 5.3.2 From observation to active participation

As novice teachers gained greater familiarity with their teaching roles, some gradually shifted from passive observers to more active participants within digital professional communities. Rather than simply consuming others' content, teachers began to share their own teaching experiences through short videos, lesson examples, and reflective posts.

*For instance, one teacher posted a short video on Douyin titled “Fun Warm-Up Games,” demonstrating how different warm-up activities could be connected through movement-based scenarios to increase students' participation. The video attracted numerous likes and comments from other teachers, many of whom highlighted its practical value and inspiration for their own teaching. Interaction records further indicate that teachers often revisited comment sections and selectively responded to peers' remarks, suggesting that they paid close attention to the feedback generated through these exchanges. (T08, digital interaction records)*

Through these interactions, digital platforms became spaces where novice teachers experimented with forms of professional self-presentation while receiving feedback from their peers. Such feedback not only provided affirmation for their instructional practices but also encouraged teachers to reflect more carefully on how their teaching ideas were perceived by others.

#### 5.3.3 Negotiating professional values through community dialogue

As teachers' participation in digital professional communities deepened, interactions on these platforms increasingly involved not only the sharing of teaching techniques but also discussions about the broader purposes and values of physical education.

These exchanges did not always lead to clear consensus. Instead, differing pedagogical perspectives and classroom approaches frequently surfaced through comments, discussions, and reflective exchanges. Through such interactions, teachers began to reconsider their own assumptions about the role of physical education in schools.

*One teacher recorded the following reflection in a teaching journal:*

“*When I first started teaching, I also thought physical education classes were just about letting students play around. Later, after seeing everyone share examples and perspectives from their actual teaching experiences, I began to wonder whether physical education classes should also shoulder the responsibility of fostering students' well-rounded development. (T13, reflection journals)”*

Through these forms of dialogue and reflection, novice teachers gradually articulated more explicit value orientations regarding their work as physical education teachers. Digital communities thus became spaces where teachers not only exchanged practical ideas but also negotiated the meaning and purpose of their professional practice.

## 6 Discussion

The findings indicate that digital informal learning serves multiple functions in novice physical education teachers' early professional lives: it operates as an orientation space amid role ambiguity, a resource for experimenting with teaching practices, and a site for articulating professional identity through digital community participation. Yet these findings also point to broader analytical questions that extend beyond functional description. In particular, further discussion is needed to explain why digital informal learning becomes a professional learning field for novice teachers, how identity is negotiated across the intersecting contexts of school and screen, and what tensions are produced as teachers move between learning, participation, and recognition in digital spaces. To address these issues, the following discussion brings social constructivist perspectives into dialogue with Bourdieu's concepts of field and habitus.

### 6.1 Digital informal learning as a professional learning field

The findings indicate that digital informal learning becomes particularly important for novice physical education teachers because it functions as an alternative professional learning field during the early stage of their careers. When entering school practice, novice teachers frequently encounter a teaching environment in which expectations remain implicit and practical guidance is limited (Ferry and Westerlund, 2023). Under these conditions, the challenge they face is not simply the acquisition of instructional techniques, but understanding how everyday teaching practices are interpreted and evaluated within the school context (Fletcher and Hordvik, 2022). Digital informal learning therefore becomes meaningful not merely as a source of teaching ideas, but as a space through which novice teachers attempt to interpret the practical logic of teaching (Staudt Willet, 2023).

A key characteristic of digital informal learning is that it expands teachers' access to observable teaching practices beyond the boundaries of their own schools. Through teaching videos, lesson demonstrations, and experience-sharing posts, novice teachers encounter multiple ways in which physical education teaching is organized, discussed, and evaluated. This exposure enables teachers to situate their own classroom experiences within a broader repertoire of professional practices rather than relying solely on the limited interpretive resources available locally (Lin and Huang, 2023). In this sense, digital informal learning functions less as a collection of ready-made strategies and more as a professional learning field in which teachers observe, interpret, and compare different enactments of teaching practice.

This process becomes clearer when both the interactional and structural dimensions of professional learning are considered. Social constructivist perspectives highlight that professional understanding develops through engagement with shared interpretations of practice (Postholm, 2012), while Bourdieu's concepts of field and habitus draw attention to the structurally weak position of novice teachers within the school field. As newcomers with limited experience and authority, they often lack the cultural and informational capital necessary to interpret the implicit rules that organize teaching practice (Mayrhofer, 2019). Digital platforms therefore operate as adjacent learning fields where teachers acquire interpretive resources that help them understand and navigate school practice (Bell-Robertson, 2014). At the same time, because visibility within digital environments is shaped by platform dynamics, certain forms of teaching practice may circulate more widely than others. Digital informal learning therefore broadens access to professional knowledge while simultaneously shaping how effective teaching is imagined.

### 6.2 Identity negotiation across school and screen

If digital informal learning constitutes a professional learning field, its influence extends beyond pedagogical knowledge to the formation of professional identity. The findings indicate that novice physical education teachers increasingly develop their sense of professional self through movement between two interconnected contexts: the institutional environment of the school and the digitally mediated spaces where teaching practices and professional experiences circulate. Professional identity therefore develops not solely within the school but through ongoing negotiation across these domains.

Within schools, novice teachers must adapt to institutional routines, classroom constraints, and organizational expectations that shape how teaching is evaluated and recognized (Betteney et al., 2018). Digital environments, however, expose teachers to a broader range of pedagogical ideas and interpretations of physical education practice. As teachers encounter alternative teaching approaches and professional perspectives online, they are prompted to reconsider how their own practices relate to these broader professional narratives. From a social constructivist standpoint, this process is significant because identity develops through participation in shared meaning-making processes. Interaction with digital communities broadens the interpretive frameworks through which teachers understand their professional roles (Dania and Griffin, 2020; Sharimova, 2025; Virta et al., 2023).

At the same time, identity negotiation across school and screen is also shaped by differing logics of recognition. In the school field, professional legitimacy is largely defined by institutional evaluation and organizational hierarchies. In digital spaces, recognition more often emerges through visibility, engagement, and the circulation of pedagogical ideas. As novice teachers participate in digital communities—sometimes by sharing teaching ideas or reflecting on classroom experiences—they may accumulate symbolic recognition among peers (Goodyear et al., 2019). Such recognition can strengthen teachers' confidence in their professional competence and encourage them to articulate their pedagogical perspectives (Richards et al., 2020; Huang et al., 2022). Yet this recognition does not necessarily translate into institutional authority within schools. Identity formation therefore becomes a negotiated process in which novice teachers continually position themselves between school-based expectations and digitally circulating models of practice.

### 6.3 Tensions in digital informal learning

Although digital informal learning provides important opportunities for professional learning and identity development, it also introduces new forms of tension for novice physical education teachers. These tensions arise because digital and institutional contexts operate according to different practical and evaluative logics.

One source of tension lies in the translation of teaching practices from digital environments into local classroom contexts. Practices that appear effective or appealing in online demonstrations are often shaped by specific contextual conditions that are not fully visible in digital representations. When novice teachers attempt to apply these ideas in their own classrooms, differences in student characteristics, teaching spaces, or institutional expectations may limit their effectiveness. Teachers therefore need to engage in substantial interpretive work to determine how digital resources can be adapted to their own teaching contexts (McNamara et al., 2019; Richards et al., 2020; McNamara et al., 2022).

A second tension concerns the dynamics of visibility and recognition within digital environments. While digital communities can function as spaces of professional exchange and support, they are also shaped by platform dynamics that privilege certain forms of participation. Practices that are visually appealing or easily shareable tend to circulate more widely, creating subtle hierarchies of symbolic recognition. Participation therefore involves not only collaboration but also comparison, as teachers become aware of how their practices are positioned relative to those of others. In this respect, social constructivist and Bourdieusian perspectives are not competing explanations but complementary ones: the former highlights the collaborative potential of digital interaction, whereas the latter draws attention to how such interaction is embedded within fields structured by unequal access to visibility and symbolic capital.

Finally, digital participation may expand the temporal and emotional demands placed on novice teachers. Continuous engagement with digital platforms—searching for resources, evaluating teaching ideas, and participating in online discussions—can extend professional learning beyond formal working hours. Digital informal learning thus expands opportunities for professional development while also intensifying the ongoing labor involved in becoming a teacher. Rather than functioning solely as a supportive learning resource, digital environments should therefore be understood as dynamic professional spaces where opportunities for learning coexist with new expectations of visibility, comparison, and continuous improvement.

## 7 Conclusions and recommendations

### 7.1 Conclusions

Drawing on qualitative data from 25 novice physical education teachers in China, the findings suggest that digital informal learning plays an important role in shaping their professional identities in the early stages of their careers. It provides an orientation space through which novice teachers interpret the uncertainties of early teaching practice, supports pedagogical experimentation as teachers selectively adapt digital resources to their own classroom contexts, and offers opportunities for participation in digital professional communities where teachers gradually articulate and negotiate their professional identities through interaction and feedback. Overall, digital informal learning expands the contexts in which novice teachers learn to become professionals. Beyond functioning as a source of teaching ideas, it enables teachers to interpret practice, develop pedagogical judgment, and position themselves within broader professional communities. In this sense, digital informal learning represents an emerging professional learning space that influences how novice physical education teachers understand their work and construct their professional identities.

### 7.2 Recommendations

To further support the role of digital informal learning in the identity development of novice physical education teachers, greater attention should be given to how schools and educational systems can create enabling conditions for such practices while avoiding excessive institutionalization. At the school level, administrators can play a constructive role by recognizing digital informal learning as a legitimate form of professional learning and by creating spaces where novice teachers are encouraged to share and reflect on digital resources and teaching experiences. Rather than transforming such practices into mandatory training or formal assessment activities, schools may incorporate informal exchange into mentoring arrangements, departmental meetings, or teacher learning activities so that digital resources can be discussed, interpreted, and adapted in relation to specific classroom contexts. In this way, school leaders can support novice teachers' engagement with digital informal learning while preserving its flexible and self-directed character.

At a broader level, educational authorities and professional organizations should also address disparities in access to digital professional resources and networks. For novice teachers working in rural or less-developed regions, limited local mentoring opportunities and narrower professional communities may restrict access to diverse teaching ideas and professional dialogue. Expanding digital infrastructure, promoting open-access repositories of high-quality teaching materials, and facilitating cross-regional online professional networks could help reduce such disparities. By connecting novice teachers across different regions and school contexts, these measures may not only broaden access to professional knowledge but also reshape the early career experience of novice physical education teachers by reducing professional isolation and strengthening their sense of professional belonging.

### 7.3 Research limitations and future prospects

Although this study strives for comprehensiveness, several limitations warrant careful consideration. First, although the sample encompasses different genders, educational levels, and regions, it remains distinctly urban-oriented overall. Among the 25 participants, there is a notable concentration in East China, which may introduce a degree of regional sampling bias. Furthermore, differences in participants' undergraduate educational backgrounds, distribution across school types, and depth of digital media usage were not systematically examined. In the Chinese educational context, digital informal learning may serve different functions in urban and rural school settings. This structural bias limits the applicability and transferability of the findings across diverse educational ecosystems. Second, although supplementary materials such as teaching reflection journals, social media posts, and digital learning interaction records were collected, the study relied primarily on teacher self-reports. It lacked more behaviorally oriented evidence, such as classroom observations or platform-level behavioral data, which limited further validation of the translation process from online learning to offline teaching. Third, the discussion of digital informal learning in this paper leans predominantly toward positive interpretations, with insufficient exploration of its potential risks. It has not systematically examined the constraining effects that platform algorithms, comparative pressures, and hidden labor may exert on teachers' professional practices and identity construction.

Future research could broaden sample composition by incorporating a wider range of school types and regional contexts, with particular attention to urban–rural differences, thereby enhancing the applicability and generalizability of findings across diverse educational settings. Additionally, integrating multiple methodological approaches—such as interviews, classroom observations, and platform-level behavioral data—is essential for providing a multidimensional understanding of teachers' digital learning practices and their translation into classroom instruction. Furthermore, subsequent research could examine how the structural mechanisms, interactional norms, and algorithmic logics of digital platforms subtly shape teachers' modes of expression and pathways of identity construction, while taking into account variations in platform preferences, usage frequency, and participation styles. This line of inquiry would foster a more balanced understanding of the complex influences shaping digital informal learning.

## Data Availability

The raw data supporting the conclusions of this article will be made available by the authors, without undue reservation.
